# Exploring the Usability and Acceptability of a Well-Being App for Adolescents Living With Type 1 Diabetes: Qualitative Study

**DOI:** 10.2196/52364

**Published:** 2023-12-22

**Authors:** Katie Garner, Hiran Thabrew, David Lim, Paul Hofman, Craig Jefferies, Anna Serlachius

**Affiliations:** 1 Department of Psychological Medicine, School of Medicine, University of Auckland, Auckland, New Zealand; 2 Liggins Institute and Department of Pediatrics, University of Auckland, Auckland, New Zealand; 3 Starship Child Health, Te Whatu Ora – Health New Zealand, Te Toka Tumai Auckland, Auckland, New Zealand

**Keywords:** well-being, digital health interventions, type 1 diabetes, diabetes, diabetic, adolescent, youth, adolescents, young people, parents, parent, mHealth, mobile health, app, apps, application, applications, acceptability, usability, interview, interviews, opinion, opinions, perception, perceptions, perspective, perspectives, acceptance

## Abstract

**Background:**

Adolescents living with either type 1 diabetes (T1D) or type 2 diabetes (T2D) have an increased risk of psychological disorders due to the demands of managing a chronic illness and the challenges of adolescence. Psychological disorders during adolescence increase the risk of suboptimal glycemic outcomes and may lead to serious diabetes-related complications. Research shows that digital health interventions may increase access to psychological support for adolescents and improve physical and mental health outcomes for youth with diabetes. To our knowledge, there are no evidence-based, publicly available mental health apps with a focus on improving the psychological well-being of adolescents with diabetes.

**Objective:**

This study aimed to explore the acceptability and usability of our evidence-based well-being app for New Zealand adolescents, *Whitu: 7 Ways in 7 Days* (*Whitu*), to allow us to further tailor it for youth with diabetes. We interviewed adolescents with T1D and T2D, their parents, and health care professionals to explore their views on the *Whitu* app and suggestions for tailoring the app for adolescent with diabetes. We also explored the cultural acceptability of the *Whitu* app for Māori and Pacific adolescents.

**Methods:**

A total of 34 participants, comprising 13 adolescents aged 12-16 years (11 with T1D and 2 with T2D), 10 parents, and 11 health care professionals, were recruited from a specialist diabetes outpatient clinic and Facebook diabetes groups. Each participant attended one 1-hour focus group on Zoom, in person, or via phone. Researchers gathered general feedback on what makes an effective and engaging app for adolescents with diabetes, as well as specific feedback about *Whitu*. Transcribed audio recordings of the focus groups were analyzed using directed content analysis.

**Results:**

Adolescents with T1D, their parents, and health care professionals found *Whitu* to be acceptable and usable. Adolescents with T1D and their parents signaled a preference for more diabetes-specific content. Health care professionals expressed less awareness and trust of digital health interventions and, as such, recommended that they be used with external support. Due to challenges in recruitment and retention, we were unable to include the views of adolescents with T2D in this qualitative study.

**Conclusions:**

There appears to be sufficient openness to the use of an app such as *Whitu* for supporting the well-being of adolescents with T1D, albeit with modifications to make its content more diabetes specific. Based on this qualitative study, we have recently developed a diabetes-specific version of *Whitu* (called *LIFT: Thriving with Diabetes*). We are also planning a qualitative study to explore the views of youth with T2D and their perspectives on the new *LIFT* app, where we are using alternative research approaches to recruit and engage adolescents with T2D and their families.

## Introduction

Adolescents living with type 1 diabetes (T1D) and type 2 diabetes (T2D) are at higher risk of psychological disorders, including anxiety, depression and eating disorders, compared to their peers without diabetes [1,2]. Such psychological disorders can increase the risk of suboptimal glycemic outcomes [3] as well as diabetes-related complications and hospitalization [4]. Although not every adolescent living with diabetes experiencing psychological distress will be diagnosed with a psychological disorder, diabetes-related psychological distress can still significantly impact their self-management and quality of life [4].

Due to factors such as time constraints and limited access to mental health services, routine psychological support is often not undertaken as part of diabetes care. Digital interventions may offer a solution for addressing this clinical gap. Research shows that digital interventions are acceptable to adolescents, cost-effective, and scalable and that they can reach populations that typically have low engagement with traditional services [5,6]. Some have even been found to be as effective as face-to-face psychological therapies [7]. However, a recent systematic review conducted by our team found limited evidence that digital interventions are effective for improving the psychological well-being of adolescents living with diabetes, highlighting the low quality of available evidence and small number of theoretically underpinned interventions [8].

*Whitu: 7 Ways in 7 Days* (*Whitu*) is a well-being app that contains seven modules to help young people (1) recognize and rate emotions, (2) learn relaxation and mindfulness, (3) practice self-compassion and (4) gratitude, (5) connect with others, (6) care for their physical health, and (7) engage in goal setting. The coping skills included in the app have all previously demonstrated efficacy for improving the well-being of young people (see our protocol paper for further information) [9]. It can be completed within a week or as desired. The app was co-designed and developed by our team together with Māori and Pacific researchers and a group of New Zealand adolescents and young adults. Previous studies with adolescents and young adults living without diabetes found that its use was associated with improved emotional and mental well-being, self-compassion, stress, sleep, depression, and anxiety at 4 weeks, with effects sustained at 3 months [10,11]. Given that *Whitu* might offer a viable solution for improving the well-being of adolescents with diabetes, this study aimed to explore the acceptability and usability of the current version of the app and to explore how it might need to be tailored for use with adolescents with T1D and T2D.

## Methods

### Study Design

A qualitative study was used. Study results are presented according to the COREQ (Consolidated Criteria for Reporting Qualitative Research) checklist [12] (see Checklist 1).

### Ethical Considerations

Ethics approval for this study was granted by the Auckland Health Research Ethics Committee (AH21899) on February 23, 2021. All participant data were deidentified after the interviews and focus groups.

### Study Participants and Recruitment

This study aimed to recruit 10 participants from each of the following groups: adolescents with T1D, adolescents with T2D, parents, and health care professionals. A total of 15 adolescents with T1D and 4 adolescents with T2D aged 12-16 years were recruited from 2 specialist pediatric diabetes clinics in Auckland, New Zealand, between April and August 2021. In addition, 3 adolescents with T1D were recruited via a Facebook advertisement. A total of 12 parents of adolescents with diabetes were recruited in clinic and 3 were recruited via a Facebook advertisement. A total of 12 health care professionals from 2 Auckland-based specialist diabetes teams were recruited via emails sent out by the first author.

Inclusion criteria for adolescents were being aged 12-16 and having been diagnosed with T1D or T2D more than 6 months ago. Exclusion criteria for adolescents included not being an English speaker or having a serious developmental or psychiatric disorder (eg, psychosis). Parents were eligible for inclusion if they were a parent of an adolescent diagnosed with T1D or T2D more than 6 months ago. Health care professionals were included if they provided care for adolescents with diabetes. During recruitment, 41 adolescents and their parents were approached to take part in the study. Of those, 38 agreed to take part in the study (24 adolescents and 15 parents) and 3 (parents) declined. Of the 20 health care professionals invited to participate via email, 11 agreed to take part in the study and 9 declined due to the lack of interest or time. Overall, 11 of the original 38 adolescents and parents were lost to follow-up, and 4 dropped out after signing informed consent. A total of 34 individuals participated in the focus groups (13 adolescents, 10 parents, and 11 health care professionals). Informed consent or assent (for adolescents aged <16 y) was obtained at recruitment, and participants were asked to fill out a baseline questionnaire regarding basic demographic information, as well as the length of diagnosis and insulin regimen for adolescents. Parents and adolescents completed consent or assent and baseline questionnaires in person at recruitment or on the web if recruited over Facebook. Health care professionals were sent a digital copy of the informed consent and baseline questionnaire.

### Focus Groups and Interviews

A total of 9 focus groups, each with 2-4 participants and lasting 30-90 minutes, were conducted over the web using Zoom (Zoom Video Communications) videoconferencing or in person using a semistructured interview schedule. Interviews over Zoom or over the phone were conducted if participants were not able to take part in the focus groups due to scheduling constraints. Adolescents with diabetes, their parents, and health care professionals participated in separate focus groups. Adolescents and their parents did not participate together. One week before the focus groups or interviews, participants received a link to download the *Whitu* app with instructions to familiarize themselves with the app before attending the focus groups or interviews. In the focus groups, the facilitators showed screenshots of each of the modules to remind the participants of the module content. Each focus group was facilitated by 2 of the following researchers: KG (female European health psychology student), Anna Boggiss (female European health psychology PhD candidate), DL (male Asian health psychology student), and Kalolaine Finaulahi (female Tongan psychology student). Six individual interviews were conducted by KG. There was no established relationship with the participants prior to study commencement. During the focus groups and interviews, participants were also asked about previous use of digital interventions. *Whitu* modules were shown to participants, and feedback on the current content, look, and feel of the app and possible diabetes-related improvements to the app was obtained. Upon completion, participants were thanked for their time and provided a gift voucher worth NZ $40 (~US $25). Audiotaped recordings were then transcribed by the first author.

### Qualitative Analysis

Transcripts were analyzed by 2 members of the research team (KG and HT) in NVivo (Lumivero) using directed content analysis, an approach where data exploration is guided by an existing framework or theory [13]. The qualitative analysis was informed by user engagement frameworks, including the Engagement, Functionality, Aesthetics, and Information domains from the user version of the Mobile Application Rating Scale [14], and used a largely deductive approach. The researchers began by independently coding the data, then forming categories, and lastly identifying descriptive themes. Any coding discrepancies were resolved by discussion with a third member of the research team (AS).

## Results

### Participant Demographics

Characteristics of the study participants can be seen in Tables 1-3 below.

**Table 1.** Demographic characteristics of the adolescents (n=13)

Characteristics		Type 1 diabetes (n=11, 85%)	Type 2 diabetes (n=2, 15%)	All adolescents (n=13)
Age (y), mean (SD)		13.6 (1.2)	12.5 (0.5)	13.4 (1.9)
**Sex, n (%)**				
	Female	8 (72)	1 (50)	9 (69)
	Male	3 (27)	1 (50)	4 (31)
**Race and ethnicity, n (%)**				
	New Zealand European	10 (91)	0 (0)	10 (77)
	Māori	1 (9)	0 (0)	1 (8)
	Pacific	2 (18)	2 (100)	4 (31)
	Chinese	1 (9)	0 (0)	1 (8)
	Other	1 (9)	0 (0)	1 (8)
Length of diabetes (y), mean (SD)		5.9 (3.52)	1 (0.5)	5.1 (3.68)
**Insulin regimen**				
	Insulin pump, n (%)	5 (46)	0 (0)	5 (38)
	Insulin injections, n (%)	5 (46)	0 (0)	5 (38)
	Medications	NovoRapid and Lantus	Lantus, NovoRapid, and Metformin	N/A^a^
**Comorbidities, n (%)**		4 (37)	1 (50)	5 (38)
	Asthma	2 (18)	0 (0)	2 (15)
	Celiac disease	1 (9)	0 (0)	1 (8)
	Graves disease	1 (9)	0 (0)	1 (8)
	High blood pressure	0 (0)	1 (50)	1 (8)

a N/A: not applicable.

**Table 2.** Demographic characteristics of the parents.

Characteristics		Parents (n=10)
Age (y) mean (SD)		47.4 (5.6)
**Sex, n (%)**		
	Female	8 (80)
	Male	2 (20)
**Race and ethnicity, n (%)**		
	New Zealand European	8 (80)
	Māori	1 (10)
	Pacific	1 (10)
	Chinese	1 (10)
**Child’s type of diabetes, n (%)**		
	Type 1	9 (90)
	Type 2	1 (10)
Reported length of child’s diabetes (y), mean (SD)		4.8 (2.71)
**Reported insulin regimen**		
	Insulin pump (children with type 1 diabetes; n=9), n (%)	4 (44)
	Insulin injections (children with type 1 diabetes; n=9), n (%)	5 (56)
	Medications	NovoRapid and Lantus
Reported comorbidities experienced by their children, n (%)		4 (40)

**Table 3.** Demographic characteristics of health care professionals.

Characteristics		Health care professionals (n=11)
Age (y), mean (SD)		43.9 (12.5)
**Sex, n (%)**		
	Female	10 (91)
	Male	1 (9)
**Race and ethnicity, n (%)**		
	New Zealand European	4 (36)
	Māori	2 (18)
	Pacific	3 (27)
	Chinese	1 (9)
	Indian	1 (9)
	Other	2 (18)
**Occupation, n (%)**		
	Diabetes nurse specialist	5 (46)
	Health psychologist	2 (18)
	Intern psychologist	1 (9)
	Community coordinator	1 (9)
	Dietitian	1 (9)
	Dietitian’s assistant	1 (9)
Years of experience, mean (SD)		12.9 (11.3)

### Qualitative Findings

#### Adolescents

Three main descriptive themes were identified from adolescents: (1) limited use of well-being–related digital interventions, (2) general acceptability of *Whitu,* and (3) a desire for more diabetes-related content to be included in the app.

##### Theme 1: Limited Use of Well-Being–Related Digital Interventions

Young people reported limited use of digital mental health interventions and reported mostly using digital tools (eg, apps) to improve their physical health and manage their diabetes (6/13, 46%). The most commonly used apps were the Freestyle Libre and xDrip+ apps to manage blood glucose levels. A smaller number (3/13, 23%) used Fitbit and other exercise apps for fitness. Only a few (2/13, 15%) adolescents said they had ever used well-being apps such as Calm and Headspace to help with relaxation and sleep. Those who reported using digital interventions to manage their physical health (6/13, 46%) noted that they were useful for managing their diabetes, fitness, or general health. Most adolescents (11/13, 85%) were open to recommending well-being apps to their friends.

*I would. To keep me on track of like, just my mental health, to keep on top of it. And like, see where I need to improve myself.* [Female adolescent, aged 15 years with T1D]

However, a few adolescents (3/13, 23%) admitted that they would feel apprehensive about how peers might perceive them. When asked if they would consider using a diabetes-related well-being app, most adolescents (11/13, 85%) agreed that they would be open to using one to improve their diabetes, better manage diabetes-related stressors, and improve their mental health.

##### Theme 2: General Acceptability of Whitu

Most adolescents (11/13, 85%) liked the look and feel of *Whitu*. Many (10/13, 77%) enjoyed the colors and graphics used in the app, and some (5/13, 38%) noted that they thought the interface was easy to use and relaxing.

*Yes. I like how it’s quite easy to follow and it’s got nice neutral colours, nice like relaxing colours.* [Male adolescent, aged 15 years with T1D]

One participant commented on the animated characters being too childish. Participants also provided feedback on ways to change the look and feel by adding more gamification or adding flowers to the *puriri* tree (the tree in the *Whitu* app that grows with each completed module). In terms of usability, most (11/13, 85%) found it easy to navigate and got used to the app quickly. One participant preferred having subtitles in the videos rather than having to play them out loud. Two adolescents said they did not like some of the videos as they were too long and became bored after watching them. Overall, adolescents liked the content of *Whitu* and found the modules and exercises useful and interesting. Some modules were more popular, with module 1 (Feel) being the most liked module and module 7 (Goal Setting) being the least liked. Regarding cultural acceptability, adolescents liked how it was linked to New Zealand and included *te reo Māori* (the Indigenous language of New Zealand). The *karanga* (traditional Māori call of welcome) at the start of the app was a highlight, and 1 participant highlighted that this made the app stand out to her.

*Yeah, I think it makes it unique and stand out from other apps, I enjoyed that part.* [Female adolescent, aged 13 years with T1D]

##### Theme 3: Desire for More Diabetes-Related Content

When asked how *Whitu* could be adapted to diabetes, most (11/13, 85%) adolescents wanted to combine diabetes management tools with well-being tools. Suggestions for possible additional content included a blood glucose tracker, dietary education focusing on carb counting, and reminders to take insulin. Many participants (9/13, 69%) also supported using the app to form connections with peers with T1D who might understand their situation and challenges.

*I think, like being able to chat to people would be quite good because then like, you can kind of relate to things and because like the camps and things those are only like an annual thing, and you don’t get to see those people quite often.* [Female adolescent, aged 12 years with T1D]

Some participants (6/13, 46%) found aspects of *Whitu* such as the badges a bit childish and suggested that it would be better to have age-specific content. Others (5/13, 38%) suggested including videos of teenagers sharing well-being or diabetes stories. A few participants (2/13, 15%) suggested that *Whitu* could include more specific information on mental health problems, such as information about self-harm.

### Parents

The four main descriptive themes identified from parents were (1) support for the use of a well-being app, (2) general acceptability of *Whitu*, (3) similar desire for more diabetes-related content, and (4) recommendations for parental involvement and support.

#### Theme 1: Support for the Use of a Well-Being App

Overall, most parents (8/10, 80%) were in favor of using a well-being app to help their children. Some parents (6/10, 60%) said they would find an app that combined diabetes management and well-being improvement highly useful. Parents reported that their kids were not using many well-being apps, if at all. However, parents expressed being willing to recommend apps to their children if they believed the apps could help their child.

*Yeah, anything like that’s going to help them. Yeah, I would definitely encourage for sure.* [Male parent of an adolescent with T1D]

#### Theme 2: General Acceptability of Whitu

*Whitu* was found to be acceptable by most parents (8/10, 80%). The app was thought to be relevant and include beneficial coping skills. One parent even described how the app had helped them and their child work through an incident at school by using the traffic light system to rate their feelings.

*We found that a really good timing and that something had happened at school that day so, especially that feelings one was a really good way of getting him to talk about what had happened.* [Female parent of an adolescent with T1D]

Other parents (4/10, 40%) said that the Relax module would be helpful to ease their child’s stress or anxiety. Parents also liked the look and feel of the app and praised the designs and colors used (6/10, 60%). Parents found the lack of subtitles on the videos inconvenient, whereas another parent commented that the app felt too stiff. Parents also reported that module 7 (Goal Setting) was repetitive and not sufficiently engaging.

#### Theme 3: Similar Desire for More Diabetes-Related Content

Similar to the adolescents, parents reported wanting more diabetes-related examples and information in the *Whitu* modules (8/10, 80%). Some parents (3/10, 30%) suggested adding information about diabetes management, including carb counting and ways to manage or track blood glucose levels. Parents also wanted to see diabetes-related examples of well-being integrated into the modules. One parent suggested that this could be done in the Be Kind to Yourself module, where self-compassion could be tied back to the challenges of diabetes management and not always having “perfect numbers” (ie, optimal hemoglobin A_1c_).

*Just making kids realise that they’re not failing if they don’t quite get it right. It’s quite important and she used to get a bit nervous going in to see the doctor because she thought, oh, what if my numbers are high and she’s a bit of a perfectionist.* [Female parent of an adolescent with T1D]

Alongside this suggestion, parents recommended the addition of encouraging phrases throughout the app and suggested there should be some way to connect teens with T1D together via the app for peer support. Suggestions to achieve the latter included web-based forums, chat functions, and diabetes groups.

#### Theme 4: Recommendations for Parental Involvement and Support

Parents noted that there was room for parental involvement in *Whitu*. Some were skeptical that their child would use the app on their own without encouragement.

*I actually think some of it might still be good with parental involvement, I don’t know how much my child would use it on their own without parents sort of instigating it.* [Female parent of an adolescent with T1D]

Others wanted the option to oversee their child’s activities on the app to facilitate conversation about areas with which they may be struggling.

### Health Care Professionals

Three main themes were identified from health care professionals: (1) limited awareness of well-being–related digital interventions, (2) variable responses to *Whitu,* and (3) some desire for clinician involvement or control.

#### Theme 1: Limited Awareness of Well-Being–Related Digital Interventions

Overall, health care professionals expressed more interest in improving their patients’ diabetes management (blood glucose tracking and diet) than their well-being via the use of digital interventions.

*Yeah, I think the more generic, the better and then the benefits will flow out into their diabetes management.* [Female diabetes nurse specialist]

Most (7/11, 64%) were unfamiliar with well-being–related and other mental health apps, expressing significant concerns about whether they would be adequate for supporting adolescents with active mental health problems. Their primary interest in well-being apps was to support the identification of “high risk” patients and timely referral for face-to-face support via the in-team psychologist and external mental health services.

#### Theme 2: Variable Responses to Whitu

Most health care professionals (8/11, 73%) spoke positively about the design and layout of *Whitu*, and many (7/11, 73%) were positive about its content. They noted that *Whitu* could increase young people’s exposure to well-being concepts, and some acknowledged the value of guided modules for learning skills such as deep breathing to aid relaxation.

*It introduces young people who might never be exposed to the possibility of doing these things, to an option where they might try it when they’re on their own in their own bedroom.* [Female diabetes nurse specialist]

Some health care professionals (5/11, 45%) were more critical about *Whitu*, especially regarding the content of the Look After Your Body module, which they thought might contradict some of the dietary advice provided to adolescents with diabetes. The Goal Setting module was also questioned as potentially being too complicated for patients to manage. Most health care professionals (8/11, 73%) misunderstood the short-term educative nature of *Whitu* (ie, to learn a suite of new skills within a week, then continue practicing preferred skills as needed) and expressed concerns about whether it would hold users’ attention on a long-term basis and be able to longitudinally track users’ progress. Finally, health care professionals (5/11, 45%) also raised concerns about discussing coping strategies without acknowledging the barriers brought on by social determinants of health and health inequities for many families living with diabetes.

*We make a lot of assumption that we all know this, so we’re all going to talk about this, but they don’t have pots and pans to cook a healthy meal.* [Female diabetes nurse specialist]

#### Theme 3: Some Desire for Clinician Involvement or Control

Health care professionals expressed some desire for involvement or control in the app. One participant suggested providing face-to-face therapy alongside some *Whitu* modules. In particular, they explained that the Look After Your Body and Goal Setting modules might be more effective if tailored to patients’ diabetes-related needs and expectations by their diabetes team.

*In terms of what I would use with my patients I would use the first five and it could work really well, alongside delivering therapy. So, you could do the same with this, but I am really worried about the look after your body and the goal setting.* [Female health psychologist]

## Discussion

### Principal Findings

Qualitative data from adolescents, parents, and health care professionals revealed important insights into perceptions of *Whitu,* and overlapping themes were identified between the 3 groups. Overall, there was sufficient acceptability of an app such as *Whitu* for supporting the well-being of adolescents with T1D. However, some modifications to the current version are required to ensure it is fit for purpose for this audience. Despite efforts to recruit and interview adolescents with T2D, insufficient data were obtained from adolescents with T2D to understand their perspectives. Further work needs to be done to improve the recruitment of this cohort.

Our finding that adolescents in this study had limited prior use of well-being apps is in line with previous research, which found that despite a quarter of adolescents being open to the idea of using mobile health (mHealth) apps, only 7.3% had ever actually used one [15]. It is likely that non–health-related technologies such as games and music are more commonly used to manage mood and stress [16]. However, this does not detract from the appeal of digital technology for digital natives, and therefore, it is encouraging that most participants were supportive of *Whitu* being adapted to be more useful for adolescents with diabetes.

The acceptability of *Whitu* by adolescents with T1D and their parents is in line with its acceptability by participants in previous studies of *Whitu* among young people without T1D [9,10]. It also aligns with previous research findings that clean, symmetrical, and creative apps are associated with better perceptions of app quality [17]. Adolescents and parents also enjoyed the content of *Whitu* and found the modules and exercises helpful and relevant. These findings echo previous qualitative studies where adolescents expressed their desire for mHealth apps to include resources relevant to their needs [16]. Although general well-being information was appreciated by adolescents, more diabetes-related content was also requested by all of them. This combination of views is consistent with previous findings that adolescents with diabetes prefer face-to-face and digital psychosocial interventions to include tailored, diabetes-specific content [18-20] and that adolescents without diabetes want well-being apps to be relevant and based on their specific needs [16]. It was reassuring to see that *Whitu* was perceived as culturally acceptable by adolescents in this study. They expressed their enjoyment of the *karanga* and the fact that the app was congruent with Māori culture and Māori models of mental health and well-being. These results highlight the importance of co-design to build tools that align with the culture and values of the app users.

Adolescents and parents expressed a preference for *Whitu* to help connect adolescents with peers who also live with diabetes. Several qualitative studies have found similar findings, where adolescents have reported that connecting with their peers was an integral part of psychosocial interventions for youth with diabetes [18,19]. This is supported by other studies in digital health where adolescents with diabetes prefer to have some form of social connection in mHealth apps [21]. Although the current version of *Whitu* has not been designed to be externally supported by parents or clinicians, it is interesting to note the findings of recent studies that peer and professional support may increase user engagement with health and well-being apps [22]. It may be that this is of greater relevance for adolescents with T1D who are more used to receiving health support than those without diabetes.

Health care professionals displayed limited familiarity with digital mental health interventions and a greater focus on the identification and management of mental health problems than the preservation or improvement of patient well-being. Given that technology including diabetes pumps and glucose-tracking apps are commonly used these days [23], this may be more a reflection of their limited familiarity with contemporary well-being interventions [24] and anxiety regarding the management of mental health problems during busy medical clinics, rather than their status as “digital immigrants” or the lack of interest in holistic patient care. Given the importance of health care professionals in the implementation of digital interventions and that digital mental health tools in diabetes should support and augment existing psychosocial support, it is essential that further iterations of *Whitu* are closely developed with this group to ensure their acceptability and feasibility within clinical settings. Formal implementation research may also need to be undertaken to maximize the impact of a diabetes-specific version of *Whitu*.

The main strength of this study was the inclusion of multiple perspectives from adolescents living with T1D, parents, and health care professionals, enabling us to gain a rich understanding of how *Whitu* might be adapted to meet the needs of this patient population.

In contrast, a limitation of this study was that we were unable to recruit any endocrinologists to explore their views of the *Whitu* app. This is somewhat mitigated by the fact that the research team includes 2 pediatric endocrinologists; however, endocrinologists represent an important stakeholder group, and their input and support of any future apps is integral to the success and implementation of well-being apps as part of diabetes care. Another limitation was our difficulty in recruiting adolescents with T2D (n=2) and their parents (n=1), which is also an issue faced by previous researchers [25]. As a result, current findings cannot be generalized to those with T2D. Given the high rate of T2D among Māori and Pacific adolescents and the lack of interventions developed for this group [8], this is an urgent gap to address in the literature. Future research in New Zealand should aim to use *Kaupapa Māori* research approaches (Māori research paradigm) that are led or coled by Māori researchers, which have shown to be highly effective for building relationships, engaging and retaining participants, and prioritizing Māori worldviews [26,27]. Other strategies to increase participant recruitment include using more culturally appropriate recruitment strategies, for example, recruiting through schools with a high percentage of Māori and Pacific students or via Māori and Pacific community groups. Cultural and language support for focus groups and greater use of face-to-face interviews may also yield more useful results. Lastly, future research should also offer adolescents with diabetes the opportunity to have individual interviews, as they may prefer to discuss potentially sensitive topics privately, without their peers present.

Based on the findings from this qualitative study, we have recently adapted *Whitu* to be a diabetes-specific well-being app called *LIFT: Thriving with Diabetes*. We are currently conducting a feasibility study of *LIFT* among youth with T1D and their parents. The adapted version contains the original 7 modules; however, they have all been tailored to be specific to diabetes. We have also added additional content to allow parents to better support adolescents and to improve parental well-being. Importantly, we are also planning a subsequent qualitative study to explore whether the newly adapted *LIFT* well-being app is engaging and useful for youth with T2D. This would allow us to explore whether *LIFT* needs to be further tailored for T2D.

### Conclusion

With diabetes-specific modifications, *LIFT* (the newly adapted version of *Whitu*) may be an acceptable digital intervention for improving the well-being of adolescents with T1D. Continued collaborative development of the app with adolescents with T2D, parents, and clinicians will be important to ensure its utility, uptake, and use in clinical care.

## Supplementary material

10.2196/52364 Checklist 1 COREQ (Consolidated Criteria for Reporting Qualitative Research) checklist.

## Abbreviations

COREQ: Consolidated Criteria for Reporting Qualitative Research
mHealth: mobile health
T1D: type 1 diabetes
T2D: type 2 diabetes
Whitu: Whitu: 7 Ways in 7 Days
